# Palmitoylation-regulated interactions of the pseudokinase calmodulin kinase-like vesicle-associated with membranes and Arc/Arg3.1

**DOI:** 10.3389/fnsyn.2022.926570

**Published:** 2022-07-28

**Authors:** Barbara Barylko, Per Niklas Hedde, Clinton A. Taylor, Derk D. Binns, Yu-Kai Huang, Gemma Molinaro, Kimberly M. Huber, David M. Jameson, Joseph P. Albanesi

**Affiliations:** ^1^Department of Pharmacology, University of Texas Southwestern Medical Center, Dallas, TX, United States; ^2^Department of Cell and Molecular Biology, John A. Burns School of Medicine, University of Hawaii, Honolulu, HI, United States; ^3^Laboratory for Fluorescence Dynamics, University of California, Irvine, Irvine, CA, United States; ^4^Department of Neuroscience, University of Texas Southwestern Medical Center, Dallas, TX, United States

**Keywords:** CaMKv, palmitoylation, pseudokinase, plasma membrane, Arc/Arg3.1, CaMKv-EGFP, Arc-mCherry

## Abstract

Calmodulin kinase-like vesicle-associated (CaMKv), a pseudokinase belonging to the Ca^2+^/calmodulin-dependent kinase family, is expressed predominantly in brain and neural tissue. It may function in synaptic strengthening during spatial learning by promoting the stabilization and enrichment of dendritic spines. At present, almost nothing is known regarding CaMKv structure and regulation. In this study we confirm prior proteomic analyses demonstrating that CaMKv is palmitoylated on Cys5. Wild-type CaMKv is enriched on the plasma membrane, but this enrichment is lost upon mutation of Cys5 to Ser. We further show that CaMKv interacts with another regulator of synaptic plasticity, Arc/Arg3.1, and that the interaction between these two proteins is weakened by mutation of the palmitoylated cysteine in CamKv.

## Introduction

Calmodulin kinase-like vesicle-associated (“CaMKv”), a member of the Ca^2+^/calmodulin-dependent protein kinase family, is almost exclusively expressed in brain and endocrine tissues (Human Protein Atlas). Because it lacks key residues required in other kinases for ATP binding, as well as the consensus autophosphorylation motif (RXXS/T) required for maintenance of CaMKII activity, CaMKv is believed to be a pseudokinase. Consistent with this view, kinase activity was not detected in *in vitro* assays (Godbout et al., 1994) and mutation of a key ATP-binding residue did not interfere with its ability to rescue activities that were lost in CaMKv knockdown neurons (Liang et al., 2016).

In a report describing CaMKv function in the nervous system, Liang et al. (2016) showed that CaMKv is critical for the maintenance of dendritic spines. They further showed that CaMKv expression is induced by sensory experience *in vivo* and by synaptic activity in cultured neurons and that overexpression of CaMKv increases spine density, whereas its depletion reduces spine density and results in impaired spatial memory. Although mechanisms underlying these synaptic functions remain to be fully elucidated, they are likely to involve regulation of the actin cytoskeleton in dendritic spines, as CaMKv was shown to interact with and inhibit the Rho guanine nucleotide exchange factor (GEF), GEF-H1, thereby suppressing RhoA activation and RhoA-dependent reduction in spine density (Liang et al., 2016).

Despite the evident importance of CaMKv in neuronal function, almost nothing is known about the protein itself. Indeed, since its discovery in 1994 by Sutcliffe’s group (Godbout et al., 1994; who named it “1G5”), only three publications focusing on CaMKv have been published (Won et al., 2006; Liang et al., 2016; Sussman et al., 2020). CaMKv was first identified as a protein associated with neuronal vesicles (Godbout et al., 1994) and was more recently found to partially localize to the plasma membrane (Liang et al., 2016; Sussman et al., 2020). However, our analysis of secondary structure prediction programs, e.g., TMHMM-2.0 (Krogh et al., 2001) and CCTOP (Dobson et al., 2015), failed to reveal a transmembrane domain. Thus, the mechanism that underlies CaMKv binding to membranes is not understood. A relatively large percentage of peripheral membrane proteins in dendrites undergo palmitoylation, the covalent modification of cysteines with 16-carbon palmitoyl chains (Zarêba-Kozioł et al., 2018). CaMKv was detected in three large-scale screens aimed at identifying palmitoylated proteins (Kang et al., 2008; Wan et al., 2013; Collins et al., 2017). In the present study, we confirm that endogenous CaMKv undergoes palmitoylation in neurons and that recombinant CaMKv undergoes palmitoylation in heterologous cells. We further show that mutation of the palmitoylation site, Cys5, results in the displacement of CaMKv from the plasma membrane. Finally, we report for the first time that CaMKv interacts directly with Arc (Activity-regulated cytoskeleton-associated protein) (Lyford et al., 1995), also known as Arg 3.1 (Activity-regulated gene 3.1) (Link et al., 1995), an activity-dependent immediate-early gene product that regulates synaptic plasticity and is required for the formation of long-term memories (Epstein and Finkbeiner, 2018; Zhang and Bramham, 2021). Our data indicate that the CaMKv-Arc interaction is influenced by CaMKv palmitoylation.

## Materials and methods

### Materials

Mouse CaMKv cDNA (Myc-DDK-tagged at the C-terminus) and mouse monoclonal anti-DDK antibody (TA50011) were from Origene. Fluorescently labeled secondary antibodies for Infrared Imaging System were from LI-COR. Cloning reagents were from Thermo Scientific. Primers and Lipofectamines were from Invitrogen. Reagents for electrophoresis and immunoblotting were from Bio-Rad. Phosphatase inhibitors cocktail (PhosSTOP) was from Roche. Thiopropyl Sepharose, reagents for analysis of palmitoylation, tissue culture, and other reagents were from Sigma.

### Generation of mutant and fluorescently-tagged constructs

Arc-mCherry construct was generated as described in Hedde et al. (2022). Fluorescently tagged CaMKv was generated by subcloning into pEGFP-C1vector (Contech) using CaMKv-Myc-DDK as a template. This CaMKv-pEGFP construct was then used as a template to generate the C5S point mutant by site-directed mutagenesis. All DNA constructs were verified by sequencing.

### Activity-regulated cytoskeleton-associated protein purification

Bacterially-expressed glutathione-S-transferase-labeled Arc (GST-Arc) was purified on Glutathione Sepharose 4B resin in buffer contained 20 mM HEPES (pH 8.0), 100 mM NaCl, 1 mM tris(2-carboxyethyl)phosphine (TCEP), 0.2 mM PMSF, and protease inhibitor cocktail (10 μg/ml each of *N*-*p*-tosyl-L-lysine chloromethyl ester, *N*-*p*-tosyl-L-arginine methyl ester, *N*-*p*-tosyl-L-lysine chloromethyl ketone, leupeptin, and pepstatin), as described (Byers et al., 2015). GST-Arc was released from the resin using 50 mM glutathione and dialyzed against 20 mM HEPES, pH 7.5, 0.1 M NaCl, 0.2 mM PMSF and 1 mM TCEP. To remove potential aggregates, Samples of Arc were centrifuged at 214,000 × *g* for 15 min at 4°C prior to all assays.

### Analysis of palmitoylation

Palmitoylation was detected using the Acyl-Resin Assisted Capture (Acyl-RAC) method (Forrester et al., 2011), as described in detail in Barylko et al. (2018). Briefly, cells or whole brain were solubilized with 2.5% SDS in 100 mM HEPES (pH 7.5), 1 mM EDTA, 0.2 mM PMSF, protease inhibitor cocktail, and 50 mM dithiothreitol (DTT). Cells were then incubated at 40°C for 0.5 h (to reduce potential S–S bonds), then for an additional 4 h with methyl methanethiosulfonate (MMTS) to block free thiols. Excess MMTS was removed by protein precipitation and washing with acetone. Dried pellets were re-solubilized in 1% SDS and mixed with thiopropyl-Sepharose resin. Half of the sample was incubated with hydroxylamine (NH_2_OH) to cleave thioester bonds, the other half was incubated with 2 M NaCl (to control for false positives). Proteins with free thiols (i.e., from cysteines that were originally palmitoylated before NH_2_OH treatment) are captured on the resin. After extensive washing, proteins released from the resin were analyzed by SDS-PAGE and identified by immunoblotting.

### GST pull-down assay of the interaction between calmodulin kinase-like vesicle-associated and activity-regulated cytoskeleton-associated protein

Calmodulin kinase-like vesicle-associated-Myc-DDK was expressed in HeLa cells and cell lysates were incubated overnight with GST-Arc or GST alone (control) bound to glutathione resin. After low-speed centrifugation (1 min at 500 × *g*), samples were washed, proteins were eluted with glutathione, and electrophoresed. CaMKv and Arc were recognized by immunoblotting with anti-DDK and anti-GST antibodies, respectively.

### Fluorescence imaging and fluorescence lifetime imaging

MCF-10A cells plated on imaging dishes coated with fibronectin were transfected with wild-type and mutant CaMKv-EGFP and Arc-mCherry using Lipofectamine 3000. Cells were imaged at room temperature 20–24 h after transfection for a maximum duration of 90 min with a Zeiss LSM880 laser scanning microscope set up for FLIM. EGFP fluorescence was excited at 880 nm (two-photon excitation, 80 MHz) and detected at 510–560 nm with a 40×, NA 1.2 water immersion lens in non-descanned mode using a hybrid photomultiplier detector (HPM-100, Becker & Hickl, Germany) coupled to a FLIMBox (ISS, Champaign, IL, United States). Per data set, 35 frames of 256 × 256 pixels were acquired with a pixel dwell time of 16 μs. Data were analyzed in SimFCS. Before FLIM, the presence of both donor (EGFP) and acceptor (mCherry) were verified with 488 and 594-nm excitation.

### Other methods

HEK293, HeLa, and MCF-10A cells (ATCC) were cultured in DMEM supplemented with 10% fetal bovine serum and antibiotics. They were transfected with either Lipofectamine 2000 (HEK293, HeLa) or Lipofectamine 3000 (MCF-10A) according to the manufacturer’s instructions and were used 20–24 h after transfection. Protein concentration was determined using the modified Lowry method according to Peterson (1977) with BSA as a standard. SDS-PAGE was carried out according to Laemmli (1970). For immunoblotting, proteins were transferred to nitrocellulose, and immunoblotted with the indicated antibodies. Bound primary antibodies were detected and quantified using fluorescently labeled secondary antibody in the LI-COR Odyssey system.

## Results

### Analysis of the putative active site of calmodulin kinase-like vesicle-associated

**FIGURE 1:**
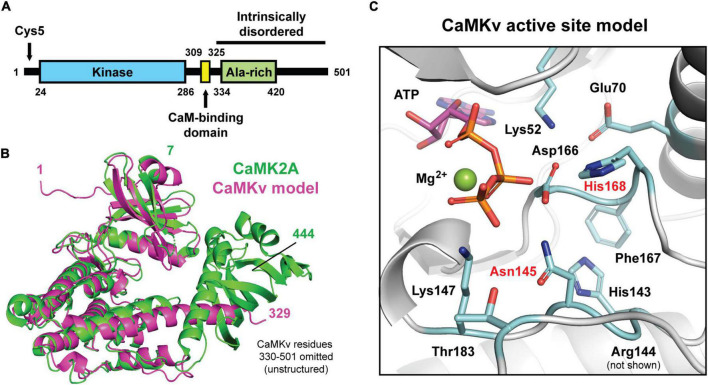
Predicted secondary and tertiary structure of calmodulin kinase-like vesicle-associated (CaMKv). **(A)** Domains of CaMKv. The intrinsically disordered region was predicted using the PONDR (Predictor of Natural Disorder Region) program. The entire sequence from residues 325–351 had PONDR scores ranging from 0.8 to 1.0. **(B)** Structural alignment (Cα r.m.s.d = 1.21 Å) of the full-length crystal structure of human CAMK2A (green; PDB: 3SOA) and a predicted model of residues 1–329 of human CaMKv derived from the AlphaFold Protein Structure Database (magenta; entry Q8NCB2). Unstructured residues 330–501 of CaMKv were omitted. **(C)** Close-up view of the predicted active site of CaMKv with modeled placement of ATP and Mg^2+^ based on an alignment to a crystal structure of CAMK2A bound to ATP (PDB: 6XBX). Residues located at positions that are important for catalysis in protein kinases are colored blue. Residues labeled in red differ from those typically found in active kinases.

Calmodulin kinase-like vesicle-associated contains an N-terminal kinase domain with 39% identity and 59% similarity to the kinase domain of CaMKIIα (CaMK2a), a central calmodulin-binding domain, and an extended C-terminal intrinsically disordered domain (∼ residues 330–501) (Figure 1A). Figure 1B shows a structural alignment of CaMK2a (obtained by X-ray crystallography) with residues 1–329 of CaMKv (predicted by AlphaFold; Jumper et al., 2021; Varadi et al., 2022). Residues T7-K300 of CaMK2a align well both structurally and by sequence with residues S18-K314 of CaMKv. However, the two proteins diverge in sequence and structure in the remaining C-terminal regions. The expanded view of the putative CaMKv active site (Figure 1C) shows that His168 replaces the glycine of the Asp-Phe-Gly (DFG) motif, which is highly conserved among kinases and which plays a critical role in catalysis. In addition, Asn145 in the catalytic loop is an aspartate in active kinases. We note that mutation of this Asp to Asn is commonly used to abolish kinase activity. Thus, it is unlikely that CaMKv expresses kinase activity.

### Palmitoylation-dependent recruitment of calmodulin kinase-like vesicle-associated to the plasma membrane

**FIGURE 2:**
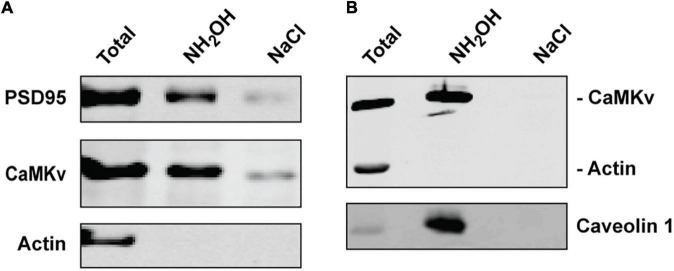
Palmitoylation of calmodulin kinase-like vesicle-associated (CaMKv). **(A)** Palmitoylation of endogenous CaMKv in mouse brain homogenate detected by Acyl-RAC (see “Materials and methods” section). PSD95 and actin are shown as positive and negative controls, respectively. Approximately 5% of input was palmitoylated in six separate experiments. **(B)** Palmitoylation of CaMKv expressed in HeLa cells. Caveolin and actin are positive and negative controls, respectively. Approximately 7% of input was palmitoylated in two separate experiments.

**FIGURE 3:**
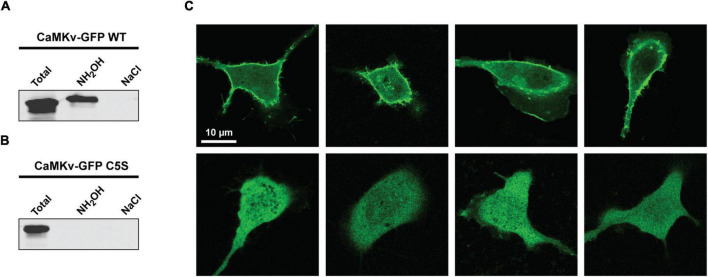
Effect of suppressing palmitoylation on the subcellular distribution of calmodulin kinase-like vesicle-associated (CaMKv). **(A,B)** Acyl-RAC analysis showing palmitoylation of wild-type CaMKv-EGFP **(A)** but not of the C5S mutant of CaMKv-EGFP in HEK-293 cells **(B)**. Approximately 4% of CaMKv-EGFP was palmitoylated in three separate experiments. **(C)** Localization of wild-type CaMKv-EGFP (top four panels) and (C5S)-CaMKv-EGFP (bottom four panels) in MCF-10A cells.

Using the Acyl-RAC approach we confirmed that endogenous CaMKv is palmitoylated in mouse brain (Figure 2A) and recombinant CaMKv is palmitoylated in HeLa cells (Figure 2B). As expected, endogenous palmitoylated CaMKv was detected in mouse brain membranes, but not in cytosolic fractions (Supplementary Figure 2A). However, only 5–10% of membrane-associated CaMKv was palmitoylated, indicating that this modification is not essential for membrane binding, but may instead contribute to specific subcellular targeting. When synaptosomal membranes were centrifuged on a sucrose step gradient, a portion of CaMKv distributed to low buoyant density fractions, commonly termed “lipid rafts,” which are enriched in palmitoylated proteins (Levental et al., 2010; Supplementary Figure 2B). To determine if palmitoylation influences the subcellular distribution of CaMKv, we localized wild-type CaMKv-EGFP and a palmitoylation-deficient CaMKv-EGFP mutant in heterologous (MCF-10) cells (Figure 3). Cysteine 5 (_1_MPFG**C**VTLGD_10_) was identified as the palmitoylation site of mouse CaMKv in a thioacylation screen (Collins et al., 2017). As shown in Figures 3A,B, wild-type CaMKv-EGFP undergoes palmitoylation in HeLa cells, whereas C5S-CaMKv-EGFP does not. In MCF-10 cells, wild-type CaMKv-EGFP shows a diffuse cytoplasmic distribution, but with a pronounced enrichment on the plasma membrane (Figure 3C, top four panels). This plasma membrane enrichment is essentially abrogated in cells expressing C5S-CaMKv-EGFP (Figure 3C, bottom four panels). During our studies, we found that the position of the EGFP tag influenced the distribution of wild-type CaMKv. Whereas CaMKv-EGFP displays pronounced plasma membrane localization in HEK-293 cells, as it does in MCF-10 cells, EGFP-CaMKv is almost entirely cytoplasmic (Supplementary Figure 1), suggesting that an N-terminal tag may suppress palmitoylation of Cys5.

### Interaction of calmodulin kinase-like vesicle-associated with activity-regulated cytoskeleton-associated protein

**FIGURE 4:**
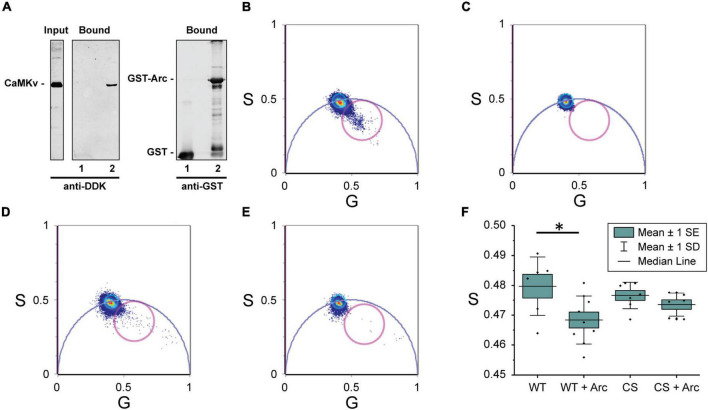
Interaction of calmodulin kinase-like vesicle-associated (CaMKv) with Arc **(A)** CaMKv binding to GST-Arc (see “Materials and methods” section). HeLa cell extract expressing CaMKv-myc-DDK was incubated with purified GST (control) or GST-Arc (10 μM) with glutathione beads. Samples were centrifuged and pelleted proteins were electrophoresed and blotted with anti-DDK or anti-GST. Lane 1: GST alone (control); Lane 2: GST-Arc. Input represents 20% of CaMKv in the incubation mixture. Similar results were obtained in three separate experiments. **(B–F)** FRET/FLIM analysis. Phasor plot pixel lifetime distribution of cells expressing: **(B)** wild-type-CaMKv-EGFP and Arc-mCherry, **(C)** wild-type CaMKv-EGFP (donor alone), **(D)** C5S-CaMKv-EGFP and Arc-mCherry, and **(E)** C5S-CaMKv-EGFP (donor alone). Points within magenta circles represent pixels in which reduction of donor lifetime (i.e., FRET) is observed. **(F)** Quantification of data in Figures 4B–E. Asterisk indicates significant difference (*p* < 0.05) using Mann-Whitney nonparametric test.

The finding that Arc binds directly to CaMKII (Donai et al., 2003) prompted us to examine whether it may also binds to CaMKv. Indeed, we found that CaMKv expressed in HeLa cells is pulled down by purified GST-Arc but not by GST alone (Figure 4A). To test whether CaMKv and Arc are likely to interact directly, we turned to measurements of Förster resonance energy transfer (FRET), which occurs if an acceptor fluorophore comes within a few nm of a donor. Energy transfer causes a reduction in both the intensity and lifetime of the donor. However, lifetime measurements are generally a more robust reporter of FRET in living cells (Padilla-Parra and Tramier, 2012). We expressed wild-type- and C5S-CaMKv-EGFP (the donor), either alone or together with Arc-mCherry (the acceptor) in MCF-10A cells and acquired lifetime images by pulsed 880 nm two-photon excitation. The fluorescence lifetime of each pixel was calculated and displayed using phasor plots (Malacrida et al., 2021; Figures 4B–E), which are 2D histograms of the pixels of cell images. In cells expressing only CaMKv-EGFP (Figures 4C,E) the center of mass of the pixel distribution falls on the universal semicircle, indicating a single exponential lifetime decay as expected for unquenched EGFP. Instead, for cells that were co-transfected with Arc-mCherry (Figures 4B,D), a comet tail extending from this region toward the zero- lifetime point (S = 0, G = 1) was observed, indicating reduced donor lifetime and, hence, the presence of FRET. Comparing the number of pixels falling outside the universal circle when Arc-mCherry is co-expressed with wild-type-CaMKv (Figure 4B) vs. the palmitoylation-deficient C5S mutant (Figure 4D), it is evident that mutation of the palmitoylation site reduces the extent of FRET between Arc and CaMKv. Quantification is provided in Figure 4F. Although palmitoylation of CaMKv apparently enhances its localization to the plasma membrane (Figure 3), we note that Arc is predominantly cytoplasmic when expressed in MCF-10A cells and that FRET between Arc-mCherry and CaMKv-EGFP was most evident in the cytoplasm (Supplementary Figure 3).

## Discussion

Here we confirm results from proteomic screens that CaMKv undergoes palmitoylation in cells and show that this modification is important for its targeting to the plasma membrane. Unlike most other forms of lipidation, palmitoylation is reversible and, hence, may be responsive to changes in neuronal conditions. Indeed, CaMKv was identified as one of 121 proteins that were differentially palmitoylated in the mouse hippocampus in response to context-dependent fear conditioning (Nasseri et al., 2021; preprint). In that study, an increase in palmitoylation of CaMKv was observed, suggesting that it may translocate to the plasma membrane upon neuronal activation. Although only about 5% of CaMKv was palmitoylated in unstimulated brain tissue, the specific pool of acylated CaMKv may play an important role in synaptic function, as was already shown for the relatively small pools of palmitoylated Arc (∼5–10%) (Barylko et al., 2018) and PICK1 (∼1%) (Thomas et al., 2013).

We also found that CaMKv interacts with Arc in a palmitoylation-sensitive manner. Arc is a positive regulator of AMPA receptor (AMPAR) endocytosis and, hence, plays a critical role in LTD. However, Arc is also required for late-stage LTP and its deletion interferes with the formation of spatial, taste, and fear memories. Like CaMKv, Arc’s promotion of late-stage LTP has been ascribed to its ability to regulate the actin cytoskeleton within dendritic spines (Bramham, 2008). There are several striking similarities between Arc and CaMKv (Liang et al., 2016). First, translation of both CaMKv and Arc is induced in dendrites in response to synaptic activity. Second, both proteins are required for late-stage LTP and spatial learning. Third, they both localize to the plasma membrane and post-synaptic density. Fourth, they both have been implicated in regulation of the actin cytoskeleton. The functional significance of the CaMKv-Arc interaction remains to be determined. We suggest that palmitoylation of Arc and CaMKv induces their colocalization to the same membrane subdomains, perhaps lipid rafts, where their coordinated activities function to regulate the postsynaptic actin cytoskeleton.

A recent report identified CaMKv as a potential immunotherapeutic target in MYCN-amplified neuroblastoma, due to its inordinately high expression in these tumors compared to normal human tissues (Sussman et al., 2020). CaMKv was found in both membrane and soluble fractions of neuroblastoma cell lines, and plasma membrane (as well as cytoplasmic) staining was detected. Notably, the study suggested that CaMKv, as a potential transmembrane protein, may be susceptible to therapeutic targeting by anti-CaMKv antibodies. However, in light of the presence of CaMKv in both membrane-bound and soluble pools, the absence of a predicted membrane-spanning motif, and our finding that palmitoylation is a likely plasma membrane targeting mechanism, it will be important to experimentally test whether or not CaMKv is indeed a transmembrane protein.

## Data availability statement

The original contributions presented in this study are included in the article/Supplementary material, further inquiries can be directed to the corresponding author.

## Author contributions

JA, BB, DJ, and KH planned and designed the study. BB, PH, CT, DB, Y-KH, and GM performed the experiments. All authors participated in writing and editing the manuscript and approved the submitted version.
