# Supplementary material for: Palmitoylation-regulated interactions of the pseudokinase calmodulin kinase-like vesicle-associated with membranes and Arc/Arg3.1
Source: Front Synaptic Neurosci. 2022 Jul 28;14:926570. doi: 10.3389/fnsyn.2022.926570 (PMC9371321; doi:10.3389/fnsyn.2022.926570)
Supplement: Supplementary file 1 [file Data_Sheet_1.docx]

Supplementary Material

# Supplementary Methods

**Preparation of synaptosomes and separation of synaptosomal membranes and cytosol.** Mouse brains were homogenized with a Dounce homogenizer in 0.31 M sucrose, 20 mM HEPES, pH 7.5, and protease inhibitors. The homogenate was centrifuged for 10 min at 1,000 x *g* and the resulting pellet was washed and centrifuged again at 1,000 x *g*. Supernatants from both spins were combined and centrifuged for 1 hour at 17,000 x *g*. The pellet, containing crude synaptosomes, was resuspended in   buffer containing 20 mM HEPES, pH 7.6, 128 mM NaCl, 3 mM KCl, 1.2 mM MgCl_2_, 0.1 mM CaCl_2_, 11 mM glucose and protease inhibitors. An aliquot of this fraction was used for palmitoylation assay, the rest was used to prepare cytosolic and membrane fractions by osmotic shock (by 10-fold dilution with 10 mM HEPES, pH 7.5) and centrifugation for 1 hour at 35,000 x *g*. Membrane fractions (pellet) were used directly for palmitoylation assay. Cytosol was concentrated, proteins were precipitated with 70% acetone, and the obtained pellet was used for palmitoylation assay.

**Preparation of low buoyant density fractions (“membrane rafts”) from mouse brain.** Synaptosomal membranes were resuspended in buffer containing 20 mM HEPES, pH 7.6, 128 mM NaCl, 3 mM KCl, 1.2 mM MgCl_2_, 0.1 mM CaCl_2_, 11 mM glucose, protease inhibitors, and 1% Triton X-100. Samples were adjusted to 42.5 % sucrose, overlaid with 35% sucrose (2 ml) and then with 16% sucrose (1.2 ml) in the synaptosomal resuspension buffer described above but without detergent. Following centrifugation at 210,000 x *g* for 20 h at 4 ⁰C in an SW60 rotor, 0.4 ml fractions were collected from the top of the tubes and equal aliquots were subjected to SDS-gel electrophoresis and immunoblotting.

# Supplementary Figures


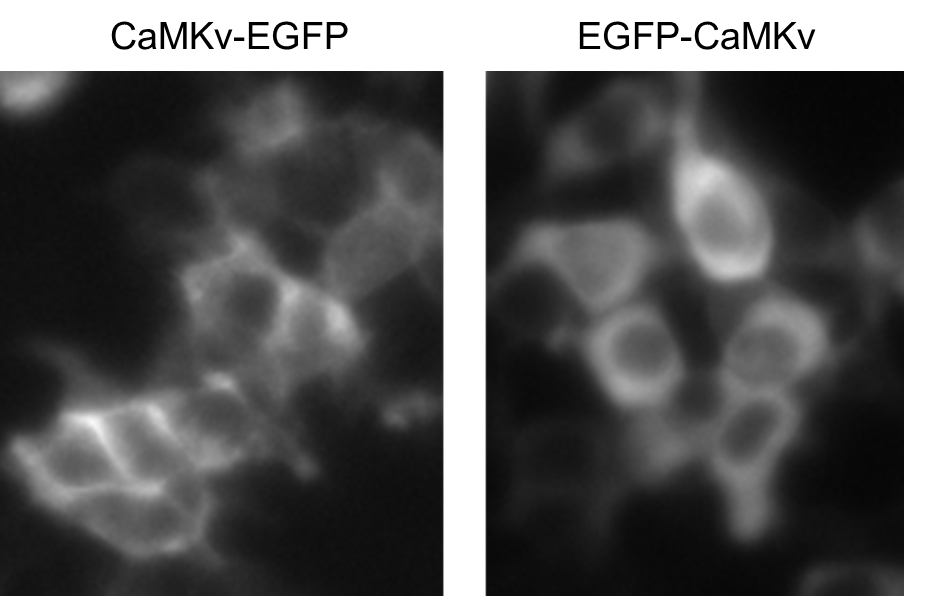


**Supplementary Figure 1.** **Distribution of CaMKv-EGFP and EGFP-CaMKv in HEK-293 cells.**

**
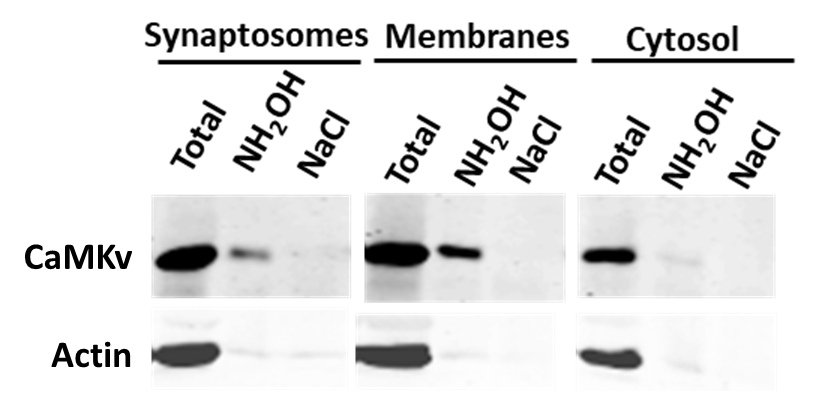
**

**Supplementary Figure 2. Selective palmitoylation of membrane-associated CaMKv in synaptosomes.** Palmitoylation of endogenous CaMKv in membrane and cytosolic fractions of mouse brain synaptosomes was detected using the Acyl-RAC procedure. Actin is presented as a negative control.

**
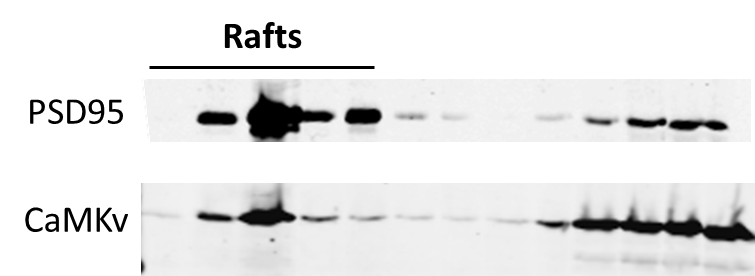
**

**Supplementary Figure 3. Association of CaMKv with low buoyant density fractions (“rafts”) from mouse brains.** Sucrose step gradient centrifugation was used to separate synaptosomal membrane fractions based on their densities. PSD95 is presented as a positive control for raft distribution.


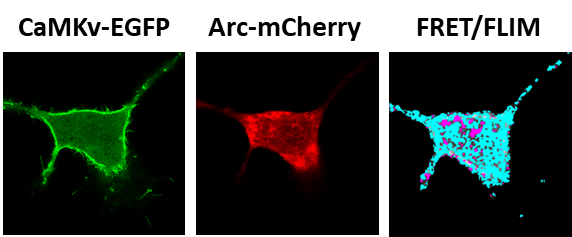


**Supplementary Figure 4. Subcellular location of FRET between Arc and CaMKv.** Figure shows a cell (imaged in Figure 3) that had been co-transfected with Arc-mCherry and analyzed for FRET/FLIM. Magenta in the right panel indicates pixels in which FRET/FLIM is evident.
